# A Lightweight Unsupervised Intrusion Detection Model Based on Variational Auto-Encoder

**DOI:** 10.3390/s23208407

**Published:** 2023-10-12

**Authors:** Yi Ren, Kanghui Feng, Fei Hu, Liangyin Chen, Yanru Chen

**Affiliations:** 1 School of Computer Science, Sichuan University, Chengdu 610065, China; renyi.scucs@gmail.com (Y.R.); fengkanghui@stu.scu.edu.cn (K.F.); hufei@stu.scu.edu.cn (F.H.); 2 Institute for Industrial Internet Research, Sichuan University, Chengdu 610065, China

**Keywords:** industrial control systems, intrusion detection, variational autoencoder

## Abstract

With the gradual integration of internet technology and the industrial control field, industrial control systems (ICSs) have begun to access public networks on a large scale. Attackers use these public network interfaces to launch frequent invasions of industrial control systems, thus resulting in equipment failure and downtime, production data leakage, and other serious harm. To ensure security, ICSs urgently need a mature intrusion detection mechanism. Most of the existing research on intrusion detection in ICSs focuses on improving the accuracy of intrusion detection, thereby ignoring the problem of limited equipment resources in industrial control environments, which makes it difficult to apply excellent intrusion detection algorithms in practice. In this study, we first use the spectral residual (SR) algorithm to process the data; we then propose the improved lightweight variational autoencoder (LVA) with autoregression to reconstruct the data, and we finally perform anomaly determination based on the permutation entropy (PE) algorithm. We construct a lightweight unsupervised intrusion detection model named LVA-SP. The model as a whole adopts a lightweight design with a simpler network structure and fewer parameters, which achieves a balance between the detection accuracy and the system resource overhead. Experimental results on the ICSs dataset show that our proposed LVA-SP model achieved an F1-score of 84.81% and has advantages in terms of time and memory overhead.

## 1. Introduction

The industrial internet is an emerging field born from the fusion of traditional industrial manufacturing and internet technology, and it is the basis for promoting the digitalization and intellectualization of industrial production, which has a broad development prospect and great strategic significance. With the development of the industrial internet, the industrial control network has shifted from closed to open, thereby giving many attackers an opportunity to exploit it. Intrusions against industrial control systems are increasing year by year. These intrusions can cause destructive consequences, such as equipment failure and downtime, production data leakage, and serious threats to personal safety, social security, and national security. In order to improve the security, stability, and robustness of ICSs, and to ensure their smooth operation, the research on intrusion detection in industrial control systems has become a hot topic in industry and academia.

Currently, the related research on intrusion detection techniques for industrial control systems can be categorized into two main categories: signature-based and anomaly-based. Signature-based intrusion detection establishes a signature library based on the intrusion behaviors that have occurred and detects them by matching [1,2]. However, signature-based methods cannot detect attacks that do not exist in the signature library and also face problems such as signature library expansion, reduced detection efficiency, and increased storage overhead, which makes it difficult to meet the needs of today’s industry for intrusion detection systems. Therefore, more research is focused on anomaly-based methods, which model normal network behavior and detect behaviors that do not conform to normal operation patterns, as well as have good recognition capabilities for unknown intrusions. Anomaly-based methods can be specifically categorized into statistical-learning-based [3,4], a-priori-knowledge-based [5], traditional machine-learning-based [6,7,8,9,10,11], and deep-learning-based methods [12,13,14,15,16,17,18,19]. Statistical-learning-based and a-priori-knowledge-based methods have advantages in intrusion detection speed; however, they cannot achieve a high detection accuracy, which may result in a large number of under-reported intrusions. Machine-learning-based methods can extract the complex features of industrial control data and achieve good intrusion detection on low-dimensional datasets, but the detection accuracy and efficiency decrease when facing large-scale high-dimensional data. Deep-learning-based methods have optimal detection performance, but if the deep learning network structure is designed to be too complex, it will result in the consumption of large amounts of computational resources and a decline in the speed of model training. Some deep-learning-based detection methods use supervised learning [12,13,14], but real industrial control data are unlabeled and require a lot of manpower for manual labeling, which limits the practical application of these methods. Therefore, some studies have proposed unsupervised deep learning detection methods [15,16,17,18,19], and these deep learning detection methods using unsupervised learning methods are consistent with the unlabeled nature of real industrial control data; they also have achieved better results on industrial control datasets. However, the focus of these existing studies is still to improve intrusion detection accuracy; the model structure is complex and has a large time and memory overhead, and it fails to fully consider the balance between intrusion detection accuracy and system resource overhead.

To solve the above problems, this study proposes a lightweight unsupervised intrusion detection model based on a variational autoencoder named LVA-SP. The model includes three stages: data processing, data reconstruction, and anomaly determination. In the data processing stage, we use the spectral residual (SR) to process the data to amplify the distribution difference between the intrusion samples and the normal samples with lightweight computation to increase the reconstruction error of the intrusion samples. In the data reconstruction stage, we propose the structure of the improved lightweight variational autoencoder (LVA), which utilizes the gate recurrent unit (GRU) with the skip mechanism and the autoregression (AR) module to improve the variational autoencoder (VAE) network, thereby skipping redundant computation and reducing the computational overhead while ensuring the accuracy of the model for intrusion detection. The anomaly determination stage uses the permutation entropy (PE) to weight the anomaly scores to further improve the detection accuracy and complete the anomaly determination. The LVA-SP model proposed in this study achieved high intrusion detection accuracy while meeting lightweight requirements.

The major contributions can be summarized as follows.

(1) In this study, we propose a lightweight unsupervised intrusion detection model based on a variational autoencoder named LVA-SP, the basic idea of which is to generate reconstructed data based on the original data and calculate the reconstruction error between the reconstructed data and the original data, as well as consider the samples with larger reconstruction errors as intrusion samples.
(2) On the industrial control dataset SWaT [20], we conducted several comparison experiments between the LVA-SP model and other baseline models, and the experimental results show that the model proposed in this study obtained the highest intrusion detection accuracy.
(3) The results of the comparison experiments show that the LVA-SP model proposed in this study has advantages in terms of time and memory overhead compared to other benchmark models.

The rest of this paper is organized as follows. Section 2 will introduce the related research work, Section 3 will elaborate on the construction of the the LVA-SP model, Section 4 will present the evaluation metrics and experimental results, and Section 5 will summarize the work of this paper.

## 2. Related Work

Being categorized by the type of detection technique, the research for intrusion detection can be divided into two main categories: signature-based methods and anomaly-based methods. The core of the signature-based intrusion detection method lies in generating signatures and establishing a signature library according to certain rules based on known attacks, wherein it detects subsequent network features by matching them in the signature library. This detection method is fast and effective in detecting known intrusions, but it cannot cope with attacks that do not exist in the signature library, i.e., the “Zero-Day Attack”. The signature library will expand with the increase in the types of intrusion behavior, thus resulting in a significant reduction in detection efficiency and an aggravation of the storage overhead and computational overhead of industrial control equipment.

The core of the anomaly-based intrusion detection method is to model the normal operation mode of the system, identify the network behaviors that do not conform to the normal operation mode, and consider these behaviors as intrusions. Compared with the signature-based approach, anomaly-based intrusion detection can solve the problem of the “Zero-Day Attack”, thereby providing industrial control systems with the ability to detect unknown attacks. In the current situation, where new types of intrusion behaviors occur frequently, signature-based intrusion detection algorithms can no longer meet the needs of current industrial control systems, so we focus on anomaly-based intrusion detection research, which is more advantageous.

### 2.1. Statistical-Learning-Based Methods

Statistical-learning-based intrusion detection methods usually record parameters such as the traffic rate, number of packets, connection rate, number of IP addresses, etc. of the network data of an industrial control system under normal conditions, and they mark the action as an intrusion when the parameter exceeds a certain threshold. Denning et al. [3] used the concept of Gaussian random variables to model a single parameter in order to better understand and characterize it, as well as to determine its threshold value. Ye et al. [4] considered the correlations between multiple parameters in order to recognize a certain class of intrusion patterns. Although the statistically based approach is very fast to compute, it has many drawbacks. First, the parameter-threshold-based judgment can be easily captured by intruders, thereby allowing intruders to easily bypass these thresholds. Second, determining thresholds is a challenging task, and it is difficult for fixed thresholds to achieve high detection accuracies. Finally, statistically based methods often rely on the assumption of a smooth processes, which is unrealistic in practical applications.

### 2.2. A-Priori-Knowledge-Based Methods

A-priori-knowledge-based intrusion detection methods require relevant domain experts to manually construct a set of rules for the normal operation of the system and establish a corresponding expert system, and behaviors that do not satisfy the rules specified by the expert system are regarded as intrusions. Estevez et al. [5] investigated an approach based on a priori knowledge, which is based on modeling network protocols and defining a series of state transitions using finite state machines, thus storing rules for legitimate patterns. This approach transitions in a finite state machine manner, thus storing rules for legitimate patterns. This type of method requires high-quality a priori knowledge and has few scenarios due to the limited number of rules stored and the high probability of false alarms.

### 2.3. Machine-Learning-Based Methods

Traditional machine-learning-based intrusion detection methods are able to mine the intrinsic features of the data and model the data, which provides better generalization ability and a higher detection accuracy compared to statistical-learning-based and a-priori-knowledge-based methods. Zhou et al. [6] proposed an intrusion detection algorithm named IBBO-LSSVM. The algorithm models and analyzes network intrusion based on support vector machines (SVMs) and applies an improved biogeography-based approach to optimize the model parameters. Liu et al. [7] addressed the resource limitation problem of wireless sensor network nodes and the lack of accuracy of intrusion detection algorithms by adopting SVM-based algorithms for intrusion detection and using improved particle swarms for parameter tuning. The results showed that the model mentioned in the paper had higher detection accuracy, faster convergence speed, and more balanced node resource utilization than other detection models. Lv et al. [8] explored the algorithm CSWC-SVM using sample weighting and category weighting to optimize the parameters of the SVM kernel function through sample weighting and category weighting, which improved the detection efficiency and accuracy. Wang et al. [9] mainly focused on one of the most frequently occurring attacks in the network, APT attacks, using a multifeature spatial-weighted combination of SVM, to capture the spatial and temporal characteristics of APT attacks and ultimately to complete the detection of this type of attack with a higher detection rate. Laskar et al. [10] combined the k-means algorithm and the isolated forest algorithm, to construct an unsupervised-learning-based intrusion detection system, to detect real-time data streams in industrial control networks. Chang et al. [11] combined the k-means algorithm and the idea of convolution to construct a clustering-based unsupervised intrusion detection system, and the results showed that the proposed method outperformed other methods on certain open-source datasets.

Although the above traditional machine learning algorithms perform well when the data volume is not large, there are limitations such as a lack of accuracy and increased overhead when dealing with large-scale high-dimensional industrial control data. For example, the commonly used SVM needs to transform the data to a higher dimensional space, which will increase the computational cost additionally. In addition, traditional machine learning algorithms require a lot of feature selection and extraction work. With the expansion of data size, traditional machine learning methods are also unable to meet the requirements of high detection accuracy.

### 2.4. Deep-Learning-Based Methods

Deep-learning-based intrusion detection methods mainly use the individual structures of deep learning networks to extract the hidden features of the data for modeling, and they feature strong generalization ability. Compared with traditional machine learning methods, they have obvious advantages in detection accuracy.

Li et al. [12] proposed a novel joint deep learning algorithm called DeepFed. The backbone structure of the algorithm consists of a convolutional neural network and a gated recurrent unit, where the convolution is used to obtain a low-dimensional feature representation of the data, and the gated recurrent unit is used to capture the temporal features of the data. Experiments on industrial control datasets have proven that the DeepFed scheme is more efficient in detecting all types of intrusions. Zhou et al. [13] proposed a twinned convolutional-neural-network-based less-sample detection model FSL-SCNN for industrial control data with few labels. The method takes the distance between the two input low-dimensional features of the twin network as the basis for determining intrusion, and, based on this, they proposed a robust loss function design containing three specific distances to improve the efficiency of the training process, mitigate the overfitting problem, and improve the accuracy of intrusion detection. Zhang et al. [14] proposed a novel convolutional neural network called RANet, which introduces grouped gated recurrent units and applies the stacking method to the last pooling layer. Compared with various state-of-the-art baseline methods, RANet improved the accuracy by about 3.21% on an open-source dataset.

Most anomaly-based deep learning methods for intrusion detection use supervised learning. However, real industrial control data is unlabeled, and a lot of manpower needs to be invested in manual labeling to provide high-quality labeled data, which leads to the limited ability of supervised methods for practical applications. Therefore, some researchers have proposed to utilize unsupervised algorithms to accomplish the task of intrusion detection. Fährmann et al. [15] proposed a variational autoencoder (VAE) with a long short-term memory (LSTM)-based model LW-LSTM-VAE to address the temporal nature of industrial control data, which introduces the LSTM into the encoding part of the VAE network and is used to capture the features of the data over time. Li et al. [16] proposed an unsupervised intrusion detection method MAD-GAN with improved generative adversarial networks (GANs). This study optimized the generation of GANs by combining LSTM and recurrent neural networks in the framework of the GANs to efficiently capture the time-series features of the data. Chen et al. [17] proposed a new unsupervised intrusion detection model, DAEMON, which is based on an autoencoder and uses a GAN structure to limit the intermediate and reconstructed outputs of the autoencoder, as well as improves model stability through adversarial training. Experimental results on four real datasets showed that the DAEMON model outperformed baseline methods. Audibert et al. [18] proposed an adversarial framework USAD; the main idea is to use two autoencoders to learn against each other for better data reconstruction and to use the reconstruction error to determine the intrusion behavior. Su et al. [19] proposed a stochastic recursive-neural-network-based method OmniAnomaly and introduced a random variable linking technique to further model the potential vector’s time-series features; finally, a dynamic threshold was introduced to determine the intrusion behavior. The method achieved good anomaly detection results on high-dimensional industrial control datasets.

The above unsupervised-based methods still focus on improving intrusion detection accuracy, with less research conducted on the simplification of the network structure and lightweight computation [21]. Among the unsupervised approaches, the generative-model-based approach is the most widely used and has good performance. Therefore, we carried out an innovative research work based on the generative mode and proposed the LVA-SP model as a result. The model adopts a lightweight design with a simpler network structure and fewer parameters.

## 3. Methodology

**Figure 1:**
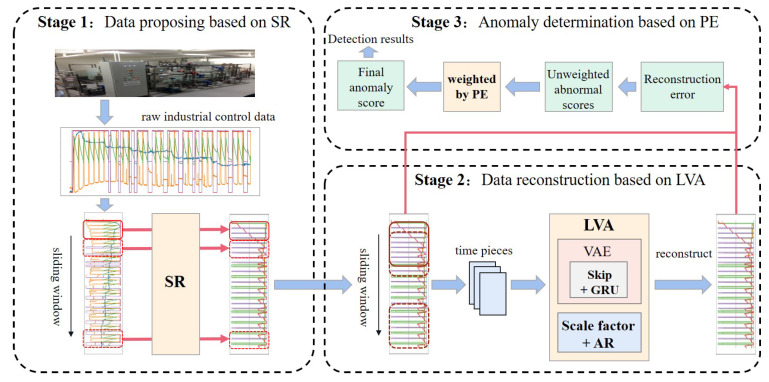
Overview of the proposed intrusion detection model LVA-SP.

This section describes the proposed intrusion detection model LVA-SP in detail. As shown in Figure 1, the model LVA-SP contains three stages.

The first stage is data processing based on spectral residuals. There may be similar potential distributions between some intrusion samples and normal samples, thereby resulting in insufficient reconstruction error of the intrusion samples, which affects the intrusion detection effect. Therefore, we propose to use SR to process the ICS data to amplify the distributional differences between the intrusion samples and the normal samples, as well as increase the reconstruction error of the intrusion samples.

The second stage is data reconstruction based on an LVA. We propose to improve the VAE by combining GRU with the skip mechanism and AR module with a scale factor, and we accordingly propose the LVA network to reconstruct the data and calculate the reconstruction error.

The third stage is anomaly determination based on PE. Each dimension of the ICS data contributes differently to the anomaly score. Therefore, we propose the ranking of the contribution based on PE as the weight of anomaly score, which is used to calculate the weighted anomaly score. Finally, the weighted anomaly score is used to determine the intrusion.

### 3.1. Data Processing Based on SR

We uses an unsupervised generative model to reconstruct the data and calculated the reconstruction error of the reconstructed data from the original data. Since the generative model tends to reconstruct normal samples, the reconstruction error of the intrusion samples tends to be larger and thus identified. However, when the generative model is more capable of reconstructing, it may also try to reconstruct the intrusion samples to reduce the reconstruction error and make the identification more difficult. This is due to the fact that intrusion samples do not differ much from normal samples in some aspects of their characteristics, thus causing the generative model to learn a latent distribution where the intrusion data is partially similar to the normal data. Therefore, when using the generative model for intrusion detection, one needs to consider processing the data accordingly to avoid unanticipated reconstructions. We used the SR to achieve this goal.

The SR is an image processing algorithm that can be used for image enhancement, image denoising, and image compression [22]. The core idea of the SR is to transform the image to the frequency domain and use the frequency domain information to detect pixels that are significantly different from the surrounding background, as is defined in the following equation:

(1) $$H_{image}=H_{innovation}+H_{priorknowledge}$$

In the SR, the $H_{image}$ is regarded as the superposition of the background part $H_{priorknowledge}$ and the salient part $H_{innovation}$. The goal is to remove the background part to retain the salient part. Upon transferring to intrusion detection, the intrusion data can be considered as the salient part of all data in a certain range. Since the waveform of the intrusion data differs from the normal data, this difference can be captured and processed by the SR in the frequency domain, thus amplifying the difference between the intrusion sample and the normal sample and increasing its reconstruction error.

The advantage of the SR is that it does not require the prior labeling or training of anomalous data, and it is therefore suitable for anomaly detection problems of unknown types, which coincides with the goal of intrusion detection tasks. In addition, the SR has the feature of a relatively lightweight computational process, which meets the overall lightweight requirement of the algorithm. By modifying the SR algorithm with intrusion detection scenarios, we propose the SR algorithm for industrial control timing data, as shown in Algorithm 1.

**Figure 2:**
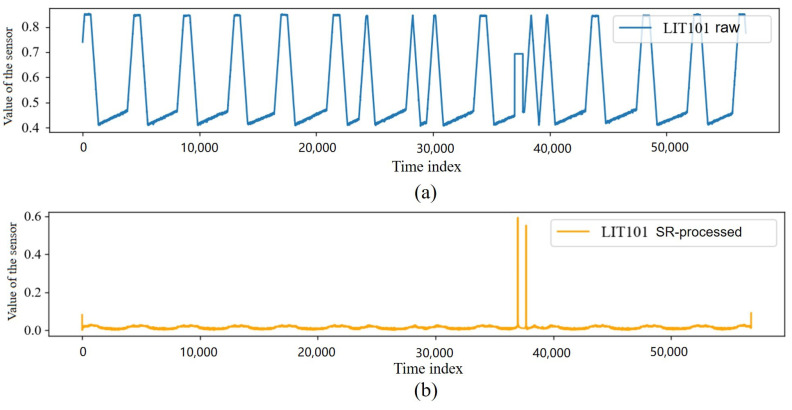
The waveform comparison of LIT101 sensor. (**a**) The raw waveform of LIT101 sensor. (**b**) The waveform of LIT101 sensor processed by SR.

We used the SR to preprocess the data in the SWaT dataset. The comparison of a sensor before and after SR processing is shown in Figure 2, which shows that the SR captured significantly different data points in the data of this sensor.
**Algorithm 1** The SR algorithm for ICS data**Input:** Raw ICS data: $X_{raw}$**Output:** ICS data processed by SR: $X_{sr}$ 1:Sampling *n* time slices with sliding step SR_shiftaccording to the sliding window size SR_w_sizeconstitutes the time slice sample set $S_{window}=${${s_{1}}^{{}^{\prime }}$, ${s_{2}}^{{}^{\prime }}$, …, ${s_{n}}^{{}^{\prime }}$} 2:$S_{sr}=\emptyset$ 3:**for** j=1→n **do**                          // *n* denotes the number of time slices 4:     $F_{j}=FFT\left({s_{j}}^{{}^{\prime }}\right)$                           // The Fourier transform 5:     $A_{j}=Amplitude\left(F_{j}\right)$              // Calculating the amplitude spectrum 6:     $P_{j}=Phrase\left(F_{j}\right)$                     // Calculation of phase spectra 7:     $AL_{j}=h_{q}\cdot A_{j}$                        // Mean filtering of the amplitude spectrum 8:     $AL_{j}=h_{q}\cdot \log\left(A_{j}\right)$        9:     $R_{j}=\log\left(A_{j}\right)-AL_{j}$                // Calculate the spectral residuals10:     $s_{j}=∥FFT^{-1}\left(\exp\left(R_{j}+iP_{j}\right)\right)∥$  // The inverse Fourier transform11:     $S_{sr}=S_{sr}\cup \left\{s_{j}\right\}$12:**end for**13:$X_{sr}\leftarrow S_{sr}$                                  // Splicing $S_{sr}$ in order14:**return** $X_{sr}$         

**Figure 3:**
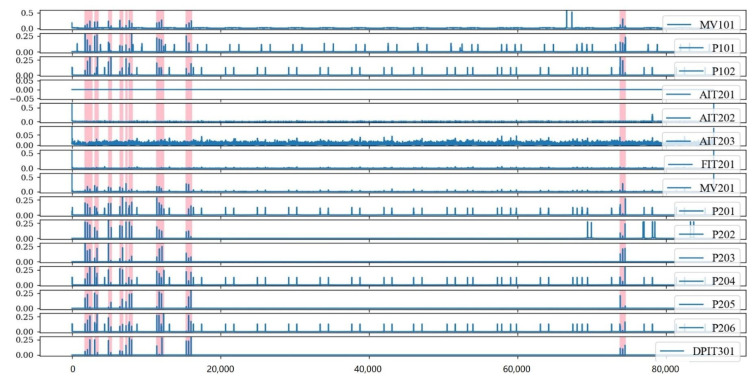
Some data of sensors processed by SR.

The timing waveforms of multiple sensors processed by the SR are shown in Figure 3, where the colored portions are the portions where intrusion behaviors occurred. In this stage, we used the SR algorithm to amplify the difference in distribution between the intrusion data and the normal data, as well as to complete the initial cleaning of the ICS data.

### 3.2. Data Reconstruction Based on LVA

Network traffic data in industrial control environments often involves hundreds of sensors, and processing these high-dimensional data causes large system resource overhead. How to realize lightweight computation for intrusion detection in industrial control scenarios while maintaining high detection accuracy is a major challenge. We propose the LVA model to solve this problem. This section describes the LVA model in five parts. The first section describes the data preprocessing. The second part describes the overall design of the LVA model. The third part introduces the improved GRU structure. The fourth part describes the reconstruction incorporating the AR module. The fifth part introduces the loss function of the LVA model.

#### 3.2.1. Data Preprocessing

To transform the raw data into a dataset that is suitable for LVA model modeling and analysis, data preprocessing is required, which consists of two main steps, which are the normalization and sliding window steps:

1. Data normalization: The normalization used in this section is the maximum and minimum value normalization, as are shown in the following equation:
  (2) $$x_{i}=\frac{x_{i}-x_{min}}{x_{max}-x_{min}}$$
2. Sliding windows are utilized to generate time window samples that can be used for model training. The window extraction step is very important, because the changes caused at the moment of the intrusion behavior may not be immediately reflected in the measured values of the sensors of the industrial control system, but they have a certain time-delay characteristics. Observation in terms of time windows takes into account this time-delay characteristic and is more in line with practical industrial control scenarios. The basic idea of window extraction is to use the sliding window method with a sliding step shift, with the length of w_size window in the time-series data gradually sliding, to divide the time-series data into several time window samples of the same length. This is shown in the following equation:
  (3) $$t_{i}=\left\{i\times shift+1,i\times shift+2,\ldots ,i\times shift+w\_size\right\}$$
  According to Equation (3), for a certain set of time-series data, the data of the (*i* + 1)th sliding window can be represented as a vector $t_{i}$ with length w_size. In order to improve the model accuracy, some overlap between neighboring windows should be allowed in the training set as much as possible to increase the number of training samples and to ensure the smoothness of the window edges so as to avoid information loss and enable the model to better understand the timing dependencies. The test set can have no overlap between neighboring windows to improve the efficiency of intrusion detection. Based on the above considerations, we set the training set shift to 1 to fully extract timing information, and we set the test set shift to w_size.

#### 3.2.2. Overall Design of LVA

The LVA model is implemented using two main modules: the VAE module and the AR module. Among them, the VAE module is mainly used for data reconstruction, which has a simpler network structure and meets the requirements of lightweight network design for industrial control intrusion detection, as well as has stronger generalization ability than a traditional autoencoder. To further ensure the lightweight nature of the network, the VAE module consists of only one layer of the encoder and one layer of the decoder.

The encoder consists of a GRU module and two dense layers. The GRU module is used to process the input time window samples, capture the hidden temporal dependencies of the samples, and output a vector of dimension. The GRU module is used to process the input time window samples, capture the hidden temporal dependencies of the samples, and output a vector of intermediate layers with a dimension of intermediate_dim. Compared with LSTM, another more commonly used module in temporal encoding [23], the GRU is more computationally efficient, because LSTM has three gates and one memory unit, while the GRU has only two gates and one hidden state, which is a lower number of parameters. At the same time, the relatively simple gating mechanism of the GRU further reduces its computation, so the GRU module can process more information in the same time step compared with LSTM, which is more satisfying for lightweight requirements. Two dense layers are used to sample the mean and variance from the intermediate layer vectors output by the GRU and variance; based on the need for backpropagation of neural networks, the sampling process is simulated using a reparameterization trick defined by the following equation:

(4) z=μ+σ⨀ε

where ε is the random variable. The final output potential variable is *z*, whose dimension is latent_dim. The activation function of the dense layer uses RELU.

The decoder consists of a GRU layer and a dense layer. The GRU reconstructs the time window samples based on time dependence, thereby converting the latent variables into an ordered set of output sequences. The dense layer is used to recover the dimensionality to that of the input sequence.

The AR module is used to add linear components to the network, thus improving the reconstruction quality. For nonlinear neural networks, when dealing with multivariate time-series data prediction problems, there is often a problem that the output scale is insensitive to the input scale [24], which leads to the prediction data not capturing some continuous local nonperiodic changes in the input data, thus affecting the prediction effect. The model in this section also suffers from the above problem in the reconstruction stage because of the nonlinearity brought on by the introduction of the RELU activation function, for which the AR module is introduced to solve this problem.

**Figure 4:**
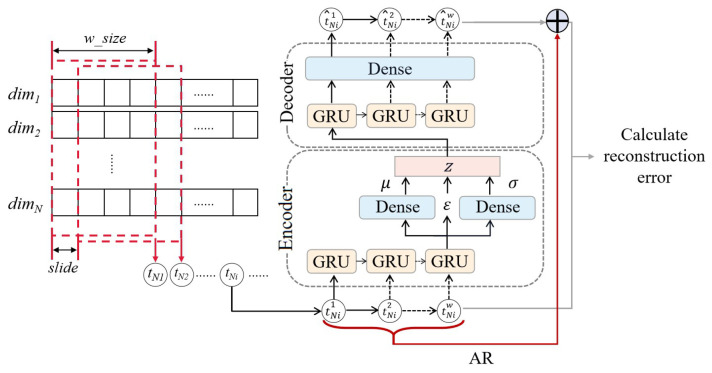
The main structure of the LVA network.

In summary, the main structure of the LVA network is shown in Figure 4.

As shown in the figure, for the *N* dimensional raw training data, the $dim_{i}$ denotes the *i*th dimension. With a sliding window of size w_size, the original data are gradually slid in the time dimension in step 1 to obtain several multidimensional time windows each of size N×w_size as the data for the input model. *w* in the Figure 4 equals to w_size, which is the size of a sliding window. For any time window sample $t_{Ni}$ input to the LVA model, $t_{Ni}=\left\{t_{Ni}^{1},t_{Ni}^{2},t_{Ni}^{3},...,t_{Ni}^{w}\right\}$. ${\hat{t}}_{Ni}$ is the reconstruction time window, and ${\hat{t}}_{Ni}^{j}$ is the *j*th data point of the reconstruction time window.

#### 3.2.3. The Improved GRU Structure

The encoder of the model in this section uses the GRU, which can capture the deep timing characteristics of industrial control data for more efficient encoding and decoding, but the original GRU has the feature of more complicated computation. To solve the above problems and reduce the computational overhead of the model, an improvement of the GRU is proposed in this section. The original computational equations of the GRU are as follows:

(5) $$r_{t}=\sigma \left(W^{r}x_{t}+U^{r}h_{t-1}\right)$$

(6) $$z_{t}=\sigma \left(W^{z}x_{t}+U^{z}h_{t-1}\right)$$

(7) $${\tilde{h}}_{t}=\tan h\left(Wx_{t}+r_{t}Uh_{t-1}\right)$$

(8) $$h_{t}=\left(1-z_{t}\right)\times h_{t-1}+z_{t}\times {\tilde{h}}_{t}$$

Among them, $z_{t}$ is the update gate, $r_{t}$ is the reset gate, $h_{t-1}$ is the output of the previous hidden layer, and $h_{t}$ is the current candidate hidden layer. $x_{t}$ is the current input data, and σ represents the activation function. *W* and *U* are the weight matrices. The update gate is used to control how the hidden state of the previous moment is combined with the input of the current moment to generate a new hidden state. If the value of the update gate is close to 0, it means that the state of the previous moment hardly needs to be updated, and if it is close to 1, it means that the state of the previous moment needs to be completely updated. The reset gate is used to control the degree of influence of the past information on the current information. When the value of the reset gate is close to 1, the current input will be strongly associated with the past information, while when the value of the reset gate is close to 0, the past information will be completely ignored, and only the current input will be used.

**Figure 5:**
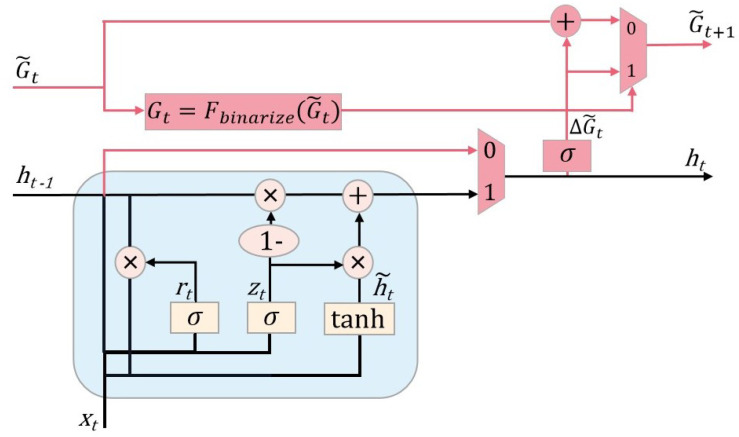
The structure of the GRU with skip mechanism.

The GRU involves a large amount of matrix computation during the operation of the update gate and reset gate, which is time consuming. In order to meet the lightweight requirements of the model, it is necessary to reduce the time consumed by the GRU during the operations. In this section, an additional binary gate structure is added to the GRU module to skip some state updates during the operation. The feasibility of skipping state updates lies in the fact that the GRU may have some redundancy in the computation of the reset and update gates in the case of processing multidimensional timing sequences [25]. The binary gate is equivalent to a skip mechanism that adaptively decides for each GRU whether it needs to update or copy from the previous time step, and when it decides to copy directly from the previous time step, it skips the tedious state update operation, thus making the model more lightweight. The modified network structure is shown in Figure 5.

${\tilde{G}}_{t}$ is the state update probability at the current moment, and $G_{t}$ is the result of the binarization of ${\tilde{G}}_{t}$. $F_{binarize}$ is the binarization function. ${\tilde{G}}_{t+1}$ is the state update probability at the next moment. $\Delta {\tilde{G}}_{t}$ is the update accumulation; whenever a state update is omitted, the state update probability of the next moment ${\tilde{G}}_{t+1}$ will be added to ${\tilde{G}}_{t}+\Delta {\tilde{G}}_{t}$, so it is not always replicated. After a state update has occurred at a certain time, the ${\tilde{G}}_{t+1}$ is refreshed to $\Delta {\tilde{G}}_{t}$ and the accumulation starts again.

With the addition of the skip mechanism, the formulas for calculating the GRU were updated as follows:

(9) $$G_{t}=F_{binarize}\left({\tilde{G}}_{t}\right)$$

(10) $$h_{t}=G_{t}\times \left[\left(1-z_{t}\right)\times h_{t-1}+z_{t}\times {\tilde{h}}_{t}\right]+\left(1-G_{t}\right)\times h_{t-1}$$

(11) $$\Delta {\tilde{G}}_{t}=\sigma \left(W\times h_{t}+b\right)$$

(12) $${\tilde{G}}_{t+1}=G_{t}\times \Delta {\tilde{G}}_{t}+\left(1-G_{t}\right)\times \left[{\tilde{G}}_{t}+\min\left(\Delta {\tilde{G}}_{t},1-{\tilde{G}}_{t}\right)\right]$$

The binarization function $F_{binarize}$, set to the round function, is as follows:

(13) $$\begin{array}{cc} \; & \mathrm{round}\left(x\right)=\left\{\begin{array}{cc} 1,\; & \mathrm{if}\;x\geq 0.5\\ 0, & \mathrm{otherwise} \end{array}\right. \end{array}$$

When setting $F_{binarize}$ to a round function, one encounters the problem of not being able to backpropagate in the network, because it is a discontinuous function, and its partial derivatives cannot be computed. The solution taken is to use a straight-through estimator (STE) [26], by setting the the bias derivative of $F_{binarize}$ to 1, so that the input gradient here in the backpropagation process is directly treated as the output gradient, thus completing the backpropagation process, which is defined as follows:

(14) $$\frac{\partial F_{binarize}\left(x\right)}{\partial x}=1$$

In summary, the GRU module with the skip mechanism added as the encoding layer is able to skip the redundant state update computation, thus speeding up the model’s computation and achieving the lightweight design.

#### 3.2.4. Reconfiguration Incombination with the AR Module

The neural network in this section is a nonlinear neural network, and nonlinear activation functions are introduced at both the GRU layer and the dense layer. Although this nonlinear characteristic increases the robustness and generalization of the neural network, it also leads to its lack of sensitivity to the local nonperiodic changes that often occur in ICS data, which affects the reconstruction effect and reduces the intrusion detection accuracy.

To address the above problems, this section proposes a further improvement of the VAE module by combining the AR module with the VAE module, which is a lightweight time-series model that is capable of linearly combining the current observations, as well as capture more time-series information in a shorter period. The AR module is used to process each time window sample, the corresponding output of the time window is calculated, and the output is called the linear component of the AR module, which is defined as follows:

(15) $$C_{i}=\sum_{j=1}^{w}W_{j}t_{Ni}^{j}+b$$

For any time window sample $t_{Ni}$, $t_{Ni}^{j}$ is the *j*th data point, $W_{j}$ is its weight, *b* is the bias term, $C_{i}$ is the computed linear component, and *w* is the size of a sliding window.

**Figure 6:**
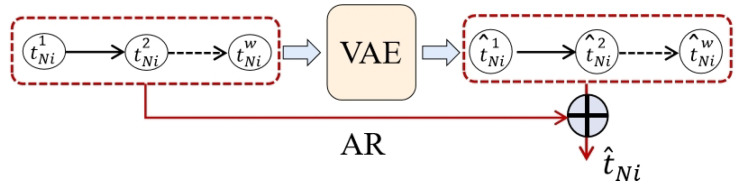
Adding the AR linear component to the output of VAE module.

Consider fitting the formula with a dense layer, which allows the model to automatically adjust the weights during the training process and finally calculate the most efficient linear component $C_{i}$. The linear components are added to the data points at each moment of the reconstructed time window sample of the VAE module using a direct addition method, and they jointly participate in the subsequent reconstruction error calculation process, as is shown in Figure 6.

$t_{Ni}$ is the original time window sample, and $t_{Ni}^{j}$ is the *j*th data point of the original time window. ${\hat{t}}_{Ni}$ is the reconstructed time window, and ${\hat{t}}_{Ni}^{j}$ is *j*th data point of the reconstructed time window. *w* is equal to w_size, which is the size of a sliding window.

To adjust the ratio between the linear component of the AR module and the nonlinear component of the VAE module, this section proposes the scale factor ρ, which is named as the AR scale factor, through which using the scale factor can adjust the ratio between the nonlinear components of the VAE output and the linear components of the autoregressive model output, which is defined as follows:

(16) $${\hat{t}}_{Ni}^{j}=\rho \times VAE\left(t_{Ni}^{j}\right)+\left(1-\rho \right)\times C_{i}$$

**Figure 7:**
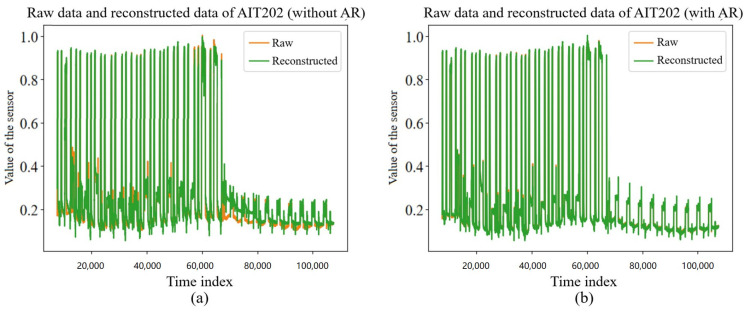
The influence of whether or not to add AR module on reconstruction. (**a**) Raw data and reconstructed data of AIT202 sensor without AR module. (**b**) Raw data and reconstructed data of AIT202 sensor with AR module.

Based on the above process, the reconstructed result combines the nonlinear component of the VAE module and the linear component of the AR module, and it is able to obtain a better quality reconstruction, as is shown in Figure 7. The left (a) of the figure shows the reconstruction without AR added, and the right (b) shows the reconstruction with AR added. It can be found that the reconstruction with AR added had better results when local nonperiodic changes occurred in the data, and it was more sensitive to such local nonperiodic changes. In addition, the computational process of AR has a very small overhead on computational resources and involves only simple linear transformations, which can improve the effectiveness of intrusion detection in a lightweight manner.

#### 3.2.5. Loss Function of LVA

The intrusion detection model based on the generative model has more stringent requirements on the quality of the data reconstruction. To improve the quality of the data reconstruction, a reasonable reconstruction loss function needs to be selected.

In VAE networks, the loss function is defined by two components: reconstruction loss and KL scatter. The KL scatter loss function is used to measure the difference between the distribution of the VAE potential variables and the standard normal distribution, and the KL scatter is continuously optimized during the training process of the network so that the distribution of the potential variables gradually approximates the standard normal distribution to improve the sampling and generation of the model, which is defined as follows:

(17) $$L_{KL}=KL(N\left(\mu ,\sigma^{2}\right)||N\left(0,1\right))$$

Reconstruction loss is used to measure the difference between the original data and the reconstructed data. For continuous-valued data such as industrial control timing data, the mean square error (MSE) is often used, which is defined as follows:

(18) $$MSE=\frac{1}{m}\sum_{i=1}^{m}{\left(y_{i}-{\hat{y}}_{i}\right)}^{2}$$

The *MSE* is more stable in gradient computation and thus more suitable for use in deep learning. Second, the *MSE* is more sensitive to outliers, because it sums the squares, thereby making the points with large errors contribute more, thus amplifying the reconstruction errors of the outliers, which is exactly in line with the scenario of industrial control intrusion detection. In this section, the *MSE* is used as the reconstruction loss function of the intrusion detection model. By combining the reconstruction error and the *KL* scatter, we used β−VAE [27] as the final loss function and assigned a weight β to the *KL* dispersion as follows:

(19) $$L=L_{MSE}+\beta \cdot L_{KL}$$

### 3.3. Anomaly Determination Based on PE

Upon the completion of the raw data reconstruction, the assessment of anomaly scores becomes essential in identifying intrusion behavior. Although many existing studies still derive anomaly scores based on the reconstruction loss utilized during network training, this method is fraught with limitations. Notably, certain intrusions exclusively target specific sensors. Consequently, while these sensors undergo significant measurement fluctuations, others might remain oblivious to the intrusion, thus resulting in considerable errors for select sensors. This scenario contrasts with the overall small error observed within the time window. Given the inadequacy of a solitary MSE metric in effectively discerning individual sensor contributions to the anomaly score, it becomes imperative to separately evaluate each sensor’s contribution [28,29,30]. We propose an anomaly determination algorithm based on PE to achieve enhanced anomaly score accuracy through a more generalized assessment.

The PE algorithm proposed in this section considers the fluctuation and complexity of the time-series signal. If the change of the time-series signal is more uncertain and random, it means that the change of its measurement value is more unpredictable, which often leads to a larger reconstruction error, so its contribution needs to be subtracted in the intrusion detection. In contrast, for stable time series with lower complexity, the model tends to reconstruct them better, which will make the reconstruction errors of the normal samples smaller, thus highlighting the reconstruction errors of intrusion samples, which is beneficial to intrusion detection, and, therefore, its contribution needs to be gained as follows:

(20) $$w_{i}\propto \frac{1}{H_{i}}$$

For the *i*th sensor, its contribution is $w_{i}$, and the measure of its timing signal complexity is $H_{i}$. *n* is the number of sensors. According to the above definition, $w_{i}$ and $H_{i}$ are inversely related.

In this section, PE is proposed as the complexity measure of the time-series signal in the above equation as *H*. PE is a metric used to analyze time series, which can quantify the randomness and complexity of the time series, as well as has the advantages of simple calculation, small time overhead, and strong noise immunity to meet the needs of lightweight models [31]. Its calculation steps are as follows:

1. For a time series $\left\{x_{1},x_{2},x_{3},\ldots ,x_{L}\right\}$ of length *L*, given an embedding dimension *m* and a time delay τ, a phase-space reconstruction is performed to obtain the matrix *Y* as follows, where d=L−(m−1)τ. Each row in the matrix *Y* represents one reconstructed component, and the total number of the reconstructed components are defined as *d*, which is shown as follows:
  (21) $$Y=\left[\begin{array}{cccc} x_{1} & x_{1+\tau } & \ldots & x_{1+(m-1)\tau }\\ x_{2} & x_{2+\tau } & \ldots & x_{2+(m-1)\tau }\\ x_{j} & x_{j+\tau } & \ldots & x_{j+(m-1)\tau }\\ \vdots & \vdots & \vdots & \vdots \\ x_{d} & x_{d+\tau } & \ldots & x_{d+(m-1)\tau } \end{array}\right]$$
2. For each reconstructed component, the index corresponding to each element is calculated after sorting in ascending order, and the index sequence of each reconstructed component is thus obtained.
3. Calculate the probability of each index sequence occurring in all index sequences $p_{j}$ and calculate the PE accordingly as follows:
  (22) $$H_{PE}=-\sum_{i=1}^{n}p_{j}\log_{2}p_{j}$$
  Subsequently, the contribution vector was calculated based on the resulting PE. After several experiments, the determined contribution degree is presented as follows:
  (23) $$w_{i}=\log\frac{1+m!}{1+H_{i}}$$
  where, for the *i*th sensor, $w_{i}$ is the contribution degree of that sensor. $H_{i}$ corresponds to $H_{PE}$ in Equation (23). *m* is the number of embedding dimensions chosen for computing the alignment entropy.

**Figure 8:**
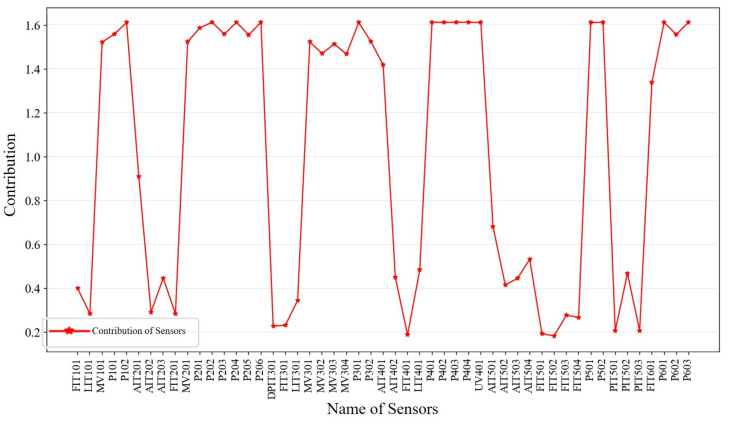
Contribution of each sensor in SWaT.

Following the above steps, we calculated the contribution of each sensor in the SWaT dataset, and the PE-based contribution reflects the stability of the sensor measurements; the results are shown in Figure 8.

The overall anomaly score for the time window is shown below using the contribution degree to weight the different sensors to the intrusion detection unit:

(24) $$Anomach\_score_{k}=\sum_{i=1}^{N}w_{i}\times score_{i}$$

where $Anomach\_score_{k}$ denotes the overall anomaly score for *k*th sample in the time windows, and $w_{i}$ is the weight of *i*th sensor. $score_{i}$ denotes the unweighted anomaly score of *i*th sensor, which is to be chosen as the MSE and the μ+σ mentioned in reference [15], which is defined as the sum of the mean and standard deviation of the difference between the reconstructed data and the input data. Finally, when the overall anomaly score of the time window is greater than the threshold, an intrusion is determined. In the algorithm of this section, the threshold value is set concerning the results obtained from the training set using the same anomaly score calculation method.

## 4. Experiments

### 4.1. Dataset and Experimental Environment

In the research of intrusion detection, Secure Water Treatment (SWaT) [20] is one of the more common industrial control datasets. This dataset was first released in 2019 by researchers from Singapore at the University of Technology and Design, and the data is derived from sensor data and network data from a small water treatment plant. SWaT contains measurements from 51 sensors with a total of 944,919 pieces of data, of which 495,000 pieces of data are for the first 7 days when the system is operating normally, and 449,919 pieces of data are for the last 4 days when it suffers from cyber attacks. In this experiment, 80% of the data of the first 7 days was used as the training dataset, which was used to fully learn the data in the normal operation mode, and the remaining 20% was used as the validation dataset. The data of the last 4 days were used as a test dataset for the detection of intrusion behavior.

The hardware environment for running the experiments in this study is shown in Table 1.

### 4.2. Operating Parameter Settings and Comparison Models

The key parameters involved in the LVA-SP model are the size of the time window w_size, the AR scale factor ρ, the middle layer size intermediate_dim, and the hidden layer size latent_dim. This section conducts experiments on w_size values of 2, 4, 8, 16, and 32, and it conducts experiments on a ρ value from 0 to 1. Finally, experiments on the intermediate_dim and latent_dim are performed for values of 16, 32, 64, and 128 to determine the optimal parameter details, as are shown in Table 2.

Among them, w_size, as the observation of the intrusion of the time window unit, should not be set too large; otherwise, it will introduce a large error to the intrusion detection. It was set to 4 to ensure that only a slight delay would be introduced. The AR scale factor ρ was set to 0.3 to achieve the best detection results, which indicates that the linear component of the data reconstruction plays a very important role. The intermediate_dim and latent_dim values should not be too large; otherwise, they will increase the model computation and memory overhead. The sliding window step shift was set to 1 in the training set to fully extract the information of the window edges, and it was set to 4 in the test set to increase the detection speed. The training epochs was set to 50, and the optimizer was chosen as Adam to increase the computation and convergence speed. The learning_rate was set to 0.001, and the batch_size was set to 512. The early stop strategy was not enabled.

In this study, we conducted comparative experiments between the proposed LVA-SP model and two commonly used unsupervised outlier detection models: KNN [16] and PCA [16]. Additionally, we compared three widely employed generative models, AE [32], VAE [32], and UAE [32], as well as three generative benchmark models: LSTM-VAE [15], USAD [18], and OmniAnomaly [19]. In this section, we individually implemented the aforementioned models and performed hyperparameter tuning for each of them to achieve optimal results.

### 4.3. Evaluation Metrics

This section describes the performance metrics used to evaluate the intrusion detection capability of the LVA-SP. Intrusion detection performance was first presented using the F1-score, which is a more comprehensive metric with high robustness to unbalanced class problems, which happens to fit the scenario where there are far more normal data than intrusion data for industrial control intrusion detection. In addition, the Precision, Recall, and Accuracy metrics were also combined. Among intrusion detection tasks, a higher Precision indicates a lower rate of model misdetection, and a higher Recall indicates a lower rate of model misses. They are calculated as follows:

(25) $$Precision=\frac{TP}{TP+FP}$$

(26) $$Recall=\frac{TP}{TP+FN}$$

(27) $$F1\text{-}score=2\cdot \frac{Precision\cdot Recall}{Precision+Recall}$$

(28) $$Accuracy=\frac{TP+TN}{TP+TN+FP+FN}$$

In the above equations, TP, FP, FN, and TN indicate the number of true positives, false positives, false negatives, and true negatives, respectively.

The receiver operating characteristic (ROC) curve and the area under the curve (AUC) were also added as evaluation criteria. The ROC curve and AUC are commonly used metrics for evaluating binary classification models. The ROC curve is a curve with the false positive rate (FPR) as the horizontal axis and the true positive rate (TPR) as the vertical axis, which indicates the classification performance of the model for the samples with true and false positive cases under different thresholds. The closer the ROC curve is to the upper left corner, the better the model performance is. The AUC is the area under the ROC curve. The larger the value, the better the model classification ability is.

In addition to the above metrics, two metrics, training time and memory overhead, were added to the experiments in this study as a basis for evaluating whether the model is lightweight or not.

### 4.4. Effectiveness of LVA-SP for Reconstructing Data

**Figure 9:**
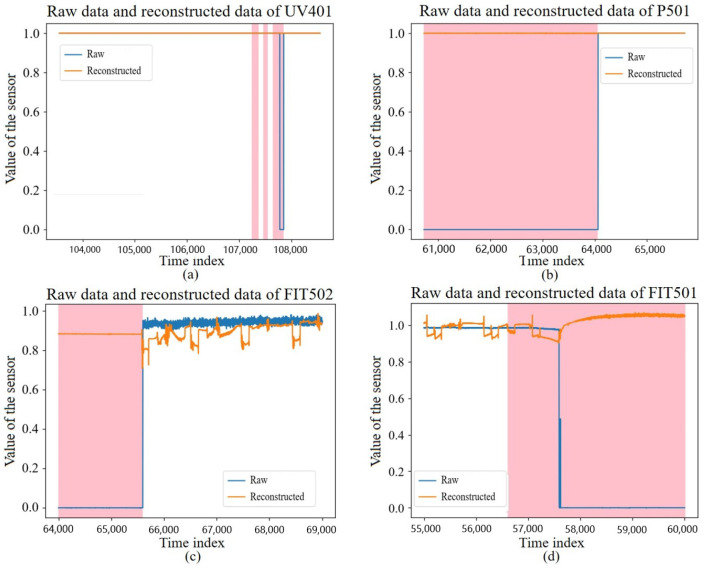
Reconstruction effect of test data of some sensors at a random time period. (**a**) Raw data and reconstructed data of UV 401 sensor. (**b**) Raw data and reconstructed data of P501 sensor. (**c**) Raw data and reconstructed data of FT502 sensor. (**d**) Raw data and reconstructed data of FT501 sensor.

The data of a sensor with a random sampling length of 5000 s from the test set is shown in Figure 9, where the colored area is the interval where the intrusion occurred. It can be found that the model could achieve good reconstruction for normal data, but it achieved poor reconstruction for intrusion data, thereby further demonstrating the feasibility of using the reconstruction error for intrusion detection.

**Figure 10:**
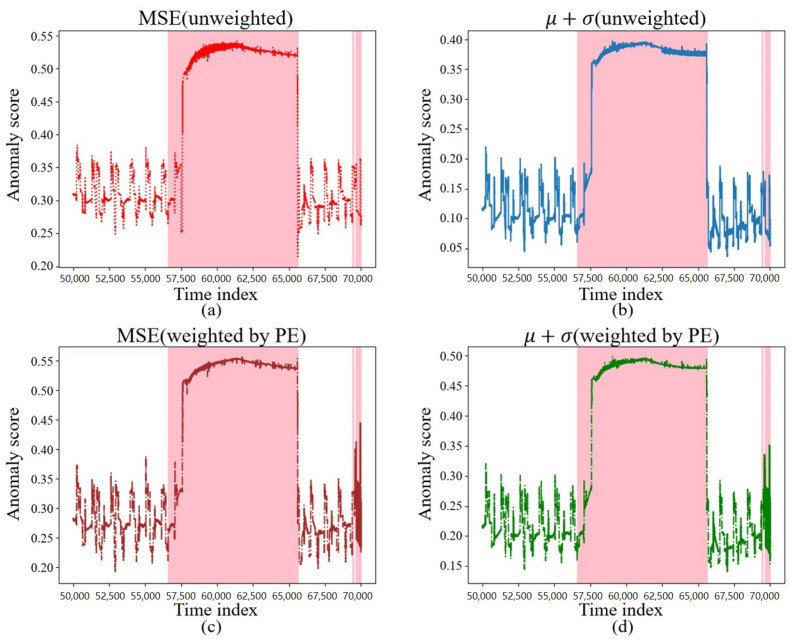
Different anomaly scores under a continuous intrusion. (**a**) Anomaly scores under MSE. (**b**) Anomaly scores under μ+σ. (**c**) Anomaly scores under MSE weighted by PE. (**d**) Anomaly scores under μ+σ weighted by PE.

**Figure 11:**
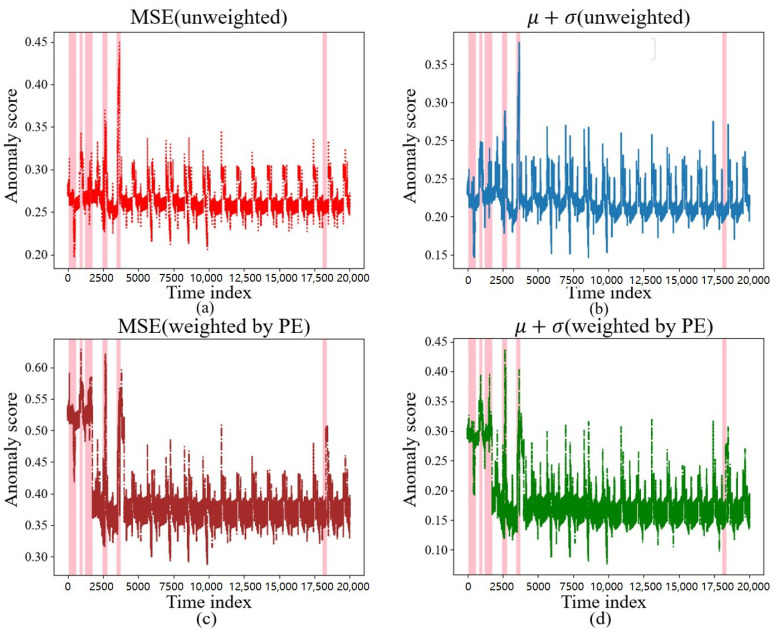
Different anomaly scores under an adjective intrusion. (**a**) Anomaly scores under MSE. (**b**) Anomaly scores under μ+σ. (**c**) Anomaly scores under MSE weighted by PE. (**d**) Anomaly scores under μ+σ weighted by PE.

The reconstruction requires the calculation of the anomaly score. In this section, we selected the unweighted MSE, μ+σ, and the PE-weighted MSE, μ+σ, for comparison, and their anomaly scores on the test data are shown in Figure 10 and Figure 11, where the colored regions are the regions where the intrusion occurred. It can be found that, during the occurrence of an intrusion of a certain duration shown in Figure 10, between the 56,500th and 65,000th time points, various different anomaly score calculation methods calculated higher anomaly scores, which could better reflect the intrusion behavior.

The anomaly score calculation method, weighted by PE, behaved more sensitively during certain short intrusion occurrences, such as between the 0th and 2500th time points, when the discrete intrusions appeared as in Figure 11, thereby further demonstrating that PE assigns greater weight to the more important sensors.

### 4.5. Ablation Experiments

In the ablation experiments, four aspects, namely, PE weighting, SR algorithm, the skip mechanism, and the AR module, were considered separately to determine the effectiveness of the method proposed in this study. The first aspect considers the effect of whether to add PE weighting on the validity of the anomaly score. The second aspect considers the effect of adding the SR algorithm on the effectiveness of the model. The third aspect considers the effect of adding the skip mechanism on the effectiveness of the model. The fourth aspect considers the effect of adding the AR module on the effectiveness of the model.

#### 4.5.1. PE Weighting

To consider the effect of PE weighting on the anomaly score, four different anomaly scores were used: MSE, μ+σ, the PE-weighted MSE, and the PE-weighted μ+σ. These four anomaly scores were evaluated on the test set using these four anomaly scores, and the results are shown in Table 3. The results show that the PE-weighted anomaly score outperformed the unweighted anomaly score on the F1-score metric, which proves that PE-weighting assigns greater weight to important sensors and is effective for intrusion detection. In addition, the PE-weighted-based MSE had the best F1-score, which is a result that is due to the fact that MSE has the property of being more sensitive to anomalies, which can further amplify the reconstruction error through the square operation. Finally, the PE-weighted MSE based on the final anomaly score was chosen to achieve an improvement of the anomaly score.

#### 4.5.2. SR Algorithm

The corresponding detection metrics are shown in Table 4, therein considering the original data directly input into the detection model and the SR-processed data input into the model. The results show that the SR-processed data had a higher F1-score, which indicates that the SR algorithm is able to bring out the intrusion features and amplify the distribution differences between the intrusion samples and normal samples, thus improving the intrusion detection accuracy based on lightweight computation.

#### 4.5.3. Skip Mechanism

The corresponding detection metrics with and without the skip mechanism are considered in Table 5. In addition to this, the time required to train 50 epochs was used as the time-overhead metric. The results show that the data processed by the skip mechanism had a higher F1-score and a 10% lower time overhead, thereby proving that the skip mechanism eliminates the redundant steps in the GRU update process.

#### 4.5.4. AR Module

The corresponding detection metrics are shown in Table 6 when considering with and without the addition of the AR module. The results show that the data processed by the AR module had a higher F1-score, which indicates that the linear component plays a key role in improving the quality of data reconstruction, and it shows that the AR module can make the model as a whole maintain a high detection accuracy while being lightweight.

**Figure 12:**
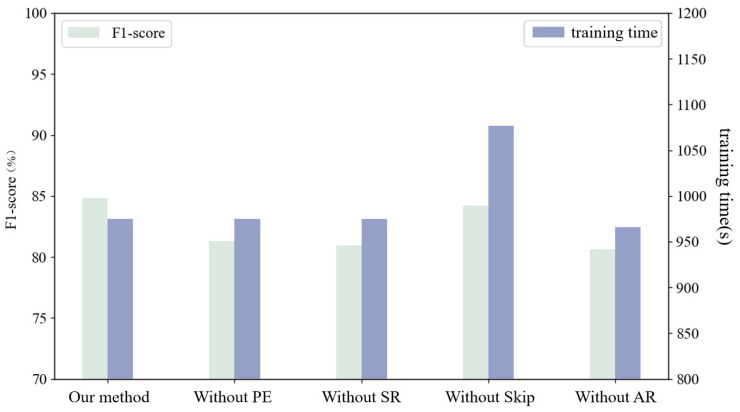
Ablation experiment of LVA-SP.

Combining the above experiments, the results of each ablation experiment are shown in Figure 12. It can be found that each module in the experiment had a positive effect on the detection accuracy and time overhead of the model. Among them, the AR module had the greatest impact on the accuracy, and the skip mechanism had the greatest impact on the time overhead.

### 4.6. Comparison Experiments

The comparison experiments will be analyzed from four perspectives, namely, detection metrics, training time, memory overhead, and detection time, where the detection metrics include the F1-score, Precision, Recall, and Accuracy comparisons. The comparison models include the KNN, PCA, AE, VAE, UAE, LSTM-VAE, USAD, and OmniAnomaly. Among them, the encode layers of the AE, VAE, and UAE modules all use simple dense layers.

#### 4.6.1. Comparison of Detection Metrics

The detection results of the above models were compared on several metrics, and the experimental results are shown in Table 7. The results show that unsupervised methods using traditional machine learning, such as KNN and PCA, did not accomplish the intrusion detection task for high-dimensional industrial control system data, and their F1-scores did not exceed 25%. Owing to their inadequate detection performance, the benchmark value was minimal, thereby leading to their exclusion from further comparsion experiments. The generative-model-based approaches performed better, with all of the F1-scores reaching 60% or more. Among them, the UAE module reached 78.54% and ran faster, but the lower recall indicates that it missed many intrusions, which limits the practical application capability.

Among the models used in the three mentioned studies, OmniAnomaly achieved the best F1-score, thereby indicating that the model has good comprehensive intrusion detection ability. USAD achieved the best Precision, thereby indicating that the model has a lower probability of determining normal data as intrusion. LSTM-VAE achieved the best Recall and the lowest Precision, which indicates that this model is a more conservative model in the intrusion detection task and tends to sacrifice a certain false detection rate to fully detect the intrusion. Upon comparing the LVA-SP model proposed in this study with the above models, it not only obtained the best F1-score, but also ranked prominently in the Precision and Recall metrics, which proves that LVA-SP has better intrusion detection ability.

**Figure 13:**
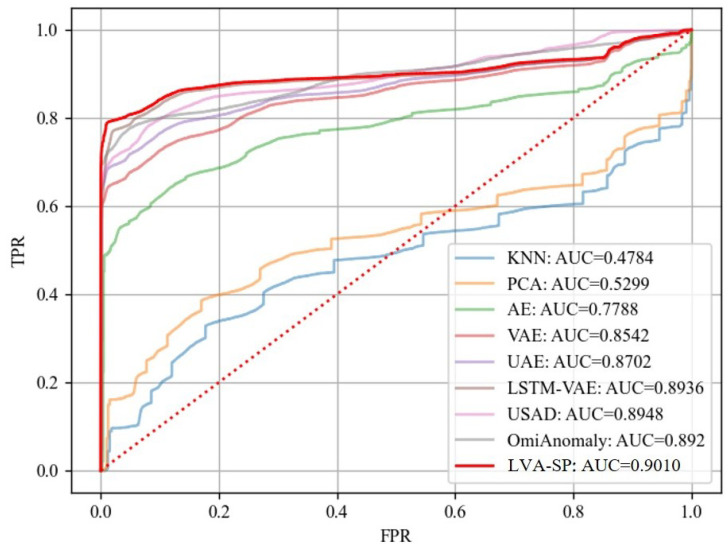
ROC curves and AUC values of all models.

The ROC curves and AUC values of all of the methods are shown in Figure 13. The ROC curves of the traditional machine learning models KNN and PCA were not satisfactory, and their AUC values were much lower than those of other comparative models, which gave a low detection performance. The generative models AE, VAE, and UAE, as well as the three benchmark models, LSTM-VAE, USAD, and OmniAnomaly, and our LVA-SP model all obtained better ROC curves and AUC values. The highlighted red line is the LVA-SP, which had the best AUC value of 0.9010. This likewise proves the superiority of the LVA-SP in terms of intrusion detection performance.

#### 4.6.2. Comparison of Training Times

**Figure 14:**
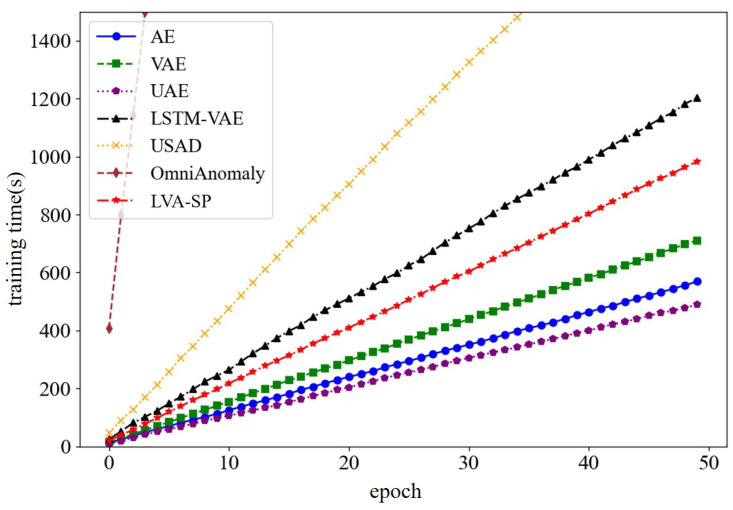
Comparison of model training times.

In comparing the training times of each of the above models, the results are shown in Figure 14. The results show that the three generative models, AE, VAE, and UAE, had the fastest training speeds, but they were far from the LVA-SP in terms of detection performance and were prone to missed or false detection of the intrusions. The LVA-SP outperformed the other three benchmark models, LSTM-VAE, USAD, and OmniAnomaly, in terms of time performance while maintaining a better detection accuracy.

#### 4.6.3. Comparison of Memory Overhead

The memory overhead considers the number of parameters used and the memory consumed by the model, as well as the memory consumed during the training process. The comparison of the above models is shown in Table 8.

Among them, the AE, VAE, and UAE models constructed based on simple dense layers have a smaller number of parameters and are smaller in terms of memory overhead. The three benchmark models LSTM-VAE, USAD, and OmniAnomaly have more parameters and higher memory overheads. The LVA-SP model has a relatively small number of parameters and minimal memory overhead compared to the three benchmark models.

#### 4.6.4. Summary of Comparison

**Figure 15:**
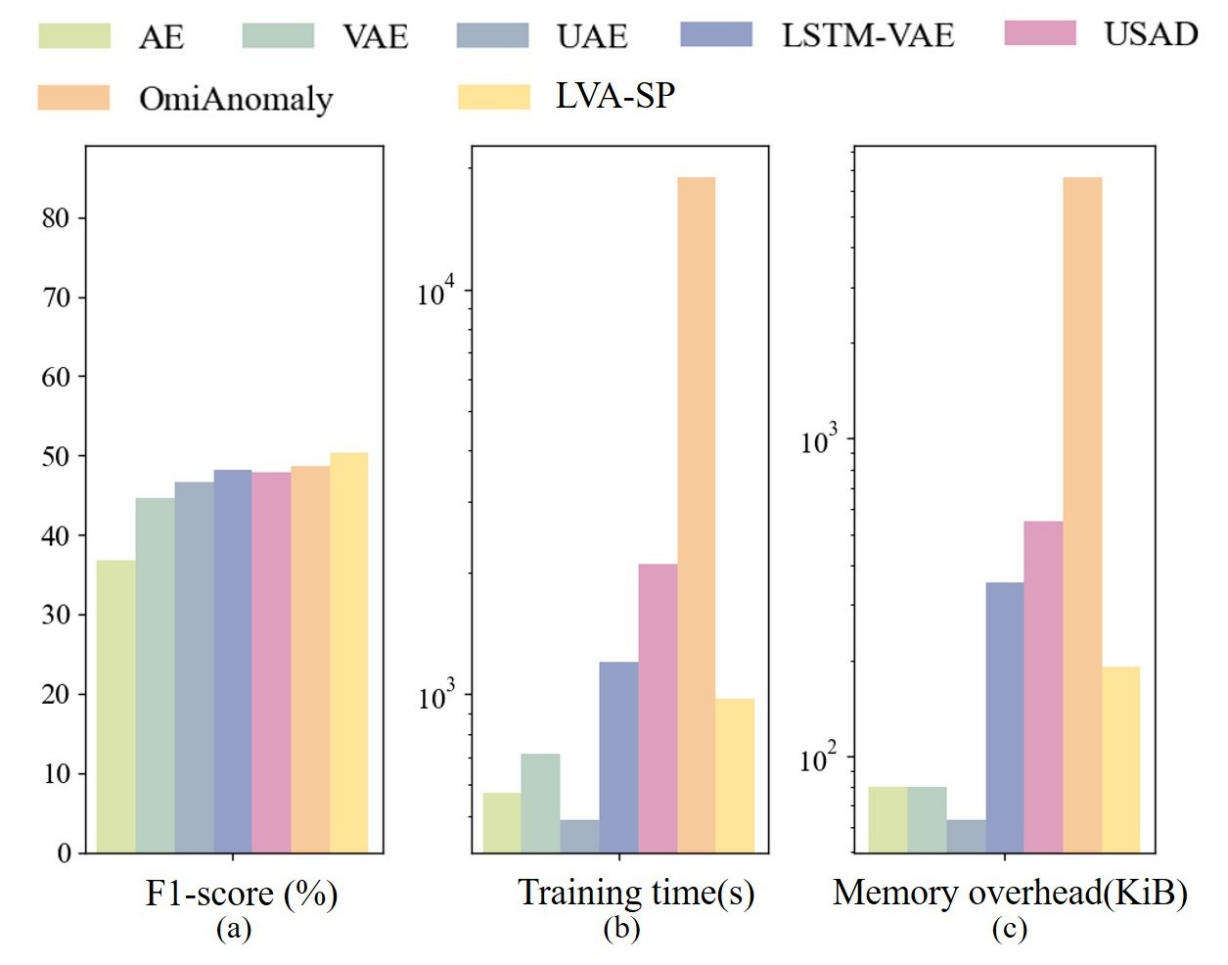
Comparison of various metrics. (**a**) Comparison of F1-score. (**b**) Comparison of training time. (**c**) Comparison of memory overhead.

Combining the F1-score, training time, and memory overhead, a comparison of the metrics of the above models is shown in Figure 15, and logarithmic vertical coordinates are used in Figure 15b,c due to the large differences in the corresponding parameter scales of the different models. The metrics show that the LVA-SP model achieved the highest F1-score and outperformed the three benchmark models of LSTM-VAE, USAD, and OmniAnomaly in terms of training time and memory overhead. Combining the detection performance, time overhead, and memory overhead, the LVA-SP model proposed in this study achieved a good balance of detection performance and resource overhead and had the highest practical application value.

## 5. Conclusions

In this study, we proposed a lightweight unsupervised intrusion detection model named LVA-SP based on the variational autoencoder. We firstly processed the data based on the SR to increase the distributional difference between the intrusion samples and the normal samples, and then we constructed the LVA network for data reconstruction, which adopted the lightweight VAE network design and improved the GRU unit in the encoder to improve the computational efficiency of the model, as well as combined the AR modulel for reconstruction. Finally, we used the anomaly score weighted by PE to determine the intrusion behavior. We conducted sufficient experiments, and the F1-score obtained by the LVA-SP reached 84.81%, which is higher than the comparative models, while there were also advantages in the time and memory overhead. The LVA-SP achieves a balance between the intrusion detection accuracy and system resource overhead.

In the future work, on the one hand, we will further improve the accuracy of the LVA-SP based on the fact that industrial control systems are frequently subjected to intrusion and have a large amount of abnormal data. On the other hand, we will conduct experiments using the LVA-SP on other public ICS datasets or using the real-time acquisition of ICS datasets to determine the generalization ability of the model. Meanwhile, in future research, we will focus on how to distinguish the anomalies caused by intrusion intentions, sensor hardware failures, etc., to further improve the performance and utility of the model and ensure its applicability in different industrial scenarios. Meanwhile, we will focus on how to distinguish the anomalies caused by intrusion, sensor hardware failures, etc., to further improve the performance and utility of the model and ensure its applicability in different industrial scenarios.

## Figures and Tables

**Table 1:** Hardware environment for experimental operation.

Type	Configuration
Processor	Intel(R) Xeon(R) Platinum 8350C CPU @ 2.60 GHz
Operating System	Ubuntu 20.04.4 LTS
Memory	32G
Video Card	GTX 3080 Ti
Video Memory	12G

**Table 2:** The parameters of the LVA-SP model.

Parameters	Value	Description
w_size	4	Sliding time window size
ρ	0.3	AR scale factor
intermediate_dim	64	Intermediate layer size
latent_dim	32	Hidden layer size
shift(train)	1	Sliding window sliding step in the training set
shift(test)	4	Sliding window sliding steps in the test set
epochs	50	Iteration rounds
optimizer	Adam	Optimizer
learning_rate	0.001	Learning Rate
batch_size	512	Batch Size

**Table 3:** Comparison of the effects of different anomaly scores.

Anomaly Score	F1-Score	Precision	Recall	Accuracy
MSE	0.8130	0.9335	0.7200	0.9596
μ+σ	0.7917	0.9685	0.6695	0.9570
MSE (PE)	0.8481	0.9249	0.7831	0.9658
μ+σ (PE)	0.8372	0.9303	0.7609	0.9628

**Table 4:** Comparison of the effect of data processing with and without SR algorithm.

With SR	F1-Score	Precision	Recall	Accuracy
Yes	0.8481	0.9249	0.7831	0.9658
No	0.8093	0.9364	0.7126	0.9462

**Table 5:** Comparison of the effect of treatment by the skip mechanism or not.

With Skip	F1-Score	Precision	Recall	Accuracy	50 Epochs of Training Time
Yes	0.8481	0.9249	0.7831	0.9658	975 s
No	0.8422	0.9236	0.7740	0.9649	1077 s

**Table 6:** Comparison of the effect of data processing with or without AR algorithm.

With AR	F1-Score	Precision	Recall	Accuracy
Yes	0.8481	0.9249	0.7831	0.9658
No	0.8061	0.9224	0.7158	0.9549

**Table 7:** Comparison of the detection metrics.

Model	F1-Score	Precision	Recall	Accuracy
KNN	0.1247	0.1610	0.1018	0.8002
PCA	0.2084	0.2922	0.1620	0.8038
AE	0.6196	0.7320	0.5371	0.9278
VAE	0.7520	0.9368	0.6281	0.9465
UAE	0.7854	0.9314	0.6789	0.9527
LSTM-VAE	0.8106	0.8619	0.7650	0.9554
USAD	0.8077	0.9475	0.7038	0.9376
OmniAnomaly	0.8205	0.9218	0.7392	0.9622
LVA-SP	0.8481	0.9249	0.7831	0.9658

**Table 8:** Comparison between the number of model parameters and the memory overhead.

Method	Number of Parameters	Memory Overhead
AE	20,627	80.57 KiB
VAE	20,627	80.57 KiB
UAE	16,133	63.02 KiB
LSTM-VAE	90,212	352.39 KiB
USAD	140,497	548.82 KiB
OmniAnomaly	1,695,135	6621.62 KiB
LVA-SP	48,760	190.47 KiB

## Data Availability

The data used to support the findings of this study are available from the corresponding author upon request.
